# Telomere length in the colon is related to colorectal adenoma prevalence

**DOI:** 10.1371/journal.pone.0205697

**Published:** 2018-10-17

**Authors:** Sarah D. Peacock, Thomas E. Massey, Stephen J. Vanner, Will D. King

**Affiliations:** 1 Department of Public Health Sciences, Queen’s University, Kingston, Ontario, Canada; 2 Department of Biomedical and Molecular Sciences, Queen’s University, Kingston, Ontario, Canada; 3 Gastrointestinal Disease Research Unit, Queen’s University, Kingston, Ontario, Canada; Tulane University Health Sciences Center, UNITED STATES

## Abstract

Telomere length has been associated with risk of several cancers. However, studies of the relationship between telomere length and colorectal cancer risk have been inconsistent. This study examined the relationship between telomere length in normal colon tissue and the prevalence of colorectal adenoma, a precursor to colorectal cancer. This nested case-control study consisted of 85 patients aged 40 to 65 undergoing a screening colonoscopy: 40 cases with adenoma(s) detected at colonoscopy and 45 controls with normal colonoscopy. During the colonoscopy, two pinch biopsies of healthy, normal appearing mucosa were obtained from the descending colon. Relative telomere length (rTL) was quantified in DNA extracted from colon mucosa using quantitative real-time PCR. Logistic regression was used to assess the relationship between telomere length and adenoma prevalence and estimate odds ratios and 95% confidence intervals. rTL was significantly longer in colon tissue of individuals with adenomas compared to healthy individuals (*p* = 0.008). When rTL was categorized into quartiles according to the distribution of rTL among controls, individuals with the longest telomeres had increased odds of adenoma when compared to individuals with shortest telomeres (OR = 4.58, 95% CI: 1.19, 17.7). This study suggests that long telomeres in normal colon tissue are associated with increased colorectal cancer risk.

## Introduction

Telomeres play an important role in maintaining chromosomal stability and genetic integrity [1,2]. The ends of each chromosome are protected by long sections of telomeric DNA consisting of variable numbers of the nucleotide sequence TTAGGG. Telomeres and their associated proteins prevent end-to-end fusion and aberrant recombination of chromosomes; also, telomeres keep genetic material from being degraded during cell divisions [3]. As a result of the end replication problem telomeres shorten with each cell division, on average by 50–100 base pairs; therefore, telomere length decreases with increasing age [4–8]. At any given age, there is substantial inter-individual telomere length variability [5,9]; this variability results from differences in genetic (e.g. telomere length heritability) [10–12], lifestyle [13–15] and environmental factors that affect telomere length [16–19].

Both short and long telomeres have been implicated in carcinogenesis. Telomere length limits uncontrolled cellular proliferation; normally, tumour suppressor mechanisms ensure that once telomeres reach a critical length, apoptosis or cellular senescence is triggered [20]. This process prevents genetic instability [2] and the accumulation of mutations [21]. Cancer cells bypass cell cycle checkpoints and continue to proliferate despite having short telomeres. Activation of the enzyme telomerase allows the cancer cell to maintain telomeres. In somatic cells, long telomeres confer higher replicative potential [21,22]. Based on the multistage model of carcinogenesis, it has been hypothesized that somatic cells that have acquired one or more initiating mutations and have constitutively long telomeres are capable of undergoing more replications and larger clonal expansion prior to the initiation of cell cycle checkpoints [21]. Therefore, compared to somatic cells with short telomeres, those with constitutively longer telomeres are at increased risk of acquiring additional genetic alterations that may ultimately lead to malignant transformation and progression [21,23–25]. Epidemiologic research into the relationship between telomere length and cancer risk generally has been inconsistent and results imply that the relationship varies by cancer site [26–30]. Many early studies indicated that shorter telomeres were associated with an increased risk for developing bladder, esophageal, gastric, and head and neck cancers [27]. More recent studies have reported that longer telomeres were associated with increased risk of several cancers including: lung, bladder, lymphoma and sarcoma [31–36].

The role of telomere length in the etiology of colorectal cancer is not well understood. Studies which have examined telomere length in blood leukocytes and colorectal cancer risk have been inconsistent, reporting no relationship [37–40], increased risk with short telomeres [39,41,42] and a U-shaped relationship [43,44]. Several studies have measured telomere length in the colorectal tissue of small numbers of patients with colorectal dysplasia or cancers; by comparing telomere length from normal colon tissue to dysplastic tissue these studies have demonstrated a tendency for altered telomere length with increasing colorectal dysplasia [45–53]. To date, only one study has compared telomere length measured in normal (healthy) colonic tissue of patients with colorectal dysplasia to the telomere length from colonic tissue of healthy individuals [40]. In that study, long telomeres were associated with the prevalence of either colorectal cancer or adenoma [40].

Colorectal tumourigenesis involves a dysplastic progression from aberrant epithelium to adenomatous polyp (adenoma) to colorectal cancer (adenocarcinoma) [54,55]. This multi-step process is characterised by increasing chromosomal and genetic instability. Adenomas, from which the vast majority of colorectal cancers arise [56,57], share the same risk factors as colorectal cancer [58]. Both the prevalence and incidence of adenomas are orders of magnitude larger than colorectal cancer prevalence and incidence [57]. Adenomas are readily detected during endoscopies, which are part of regular population-level surveillance programs for colorectal cancer. Experimental and observational studies support the use of adenomas as a surrogate endpoint for colorectal cancer [59–62].

In summary, the role of telomere length in colorectal carcinogenesis warrants further examination as results from studies of the association between telomere length and colorectal cancer risk remain equivocal. Within an individual, telomere length varies across tissues [8]; thus, investigations of telomere length in healthy colon tissue in relation to colorectal cancer risk are needed to inform on this relationship. As telomere length may be related to early events in the adenoma-carcinoma sequence, adenomas are an informative endpoint for studies of colorectal cancer risk. This study examined the relationship between telomere length in normal colonic mucosa and the prevalence of colorectal adenoma.

## Materials and methods

### Study population

This case-control study was nested in a cross-sectional study which recruited patients undergoing a screening colonoscopy at a regional endoscopy centre in Kingston, Ontario, Canada. Screening colonoscopies were performed on patients with a positive family history of colorectal adenoma or cancer, patients who had a positive fecal occult blood test (FOBT), and patients at average risk for colorectal cancer. Between 2009 and 2012, healthy male and female colonoscopy patients aged 40 to 65 were invited to participate in the cross-sectional study (patients with the following conditions were not invited to participate: inflammatory bowel disease, genetic disorders that predispose to colorectal cancer, any gastrointestinal abnormality detected at a previous colonoscopy, a diagnosis of cancer within the previous 5 years). Participants in the cross-sectional study consisted of 444 patients who provided written informed consent. Complete study design details and subject eligibility and exclusion criteria can be found elsewhere [63].

Briefly, consenting patients were asked to complete a self-administered research questionnaire prior to their colonoscopy. At the time of colonoscopy a fasting blood sample and two pinch biopsies of colonic tissue were collected for biochemical and genetic analyses. Biopsies were obtained from healthy, normal appearing mucosa in the descending colon at least 10 cm from any abnormalities and the two biopsies were taken 10 cm apart from one another. Anthropometric data and colonoscopy results were obtained from medical records. The study was approved by the Queen's University Health Science Research Ethics Board.

Of the 444 patients who consented to participate in the cross-sectional study, 48 were excluded due to incomplete colonoscopies, 50 were excluded due to a diagnosis of colorectal cancer, hyperplastic polyps or inflammatory bowel disease; a further 12 participants were excluded as a result of insufficient DNA for analyses. The final sample available for this study consisted of 334 patients.

### Selection of cases and controls

Participants were selected for this case-control study based on the pathology reports of the screening colonoscopy. Any colorectal abnormality detected during colonoscopy was removed and assessed by a gastrointestinal pathologist using standard diagnostic criteria. The case group consisted of patients who were found to have one or more colorectal adenomas at colonoscopy. Of the 110 patients with adenomas, 40 were randomly selected as the case group for this study. Patients with no colorectal abnormalities detected at colonoscopy comprised the control group. Of the 224 patients with normal colonoscopy, 45 were randomly selected as controls for this study.

### Measurement of relative telomere length (rTL)

Colonic tissue samples obtained from each participant were placed immediately into 5 Prime cell lysis solution (Intermedico) and stored at -20°C until DNA extraction. Genomic DNA was isolated from colonic tissue using 5 Prime ArchivePure DNA Tissue Kits (Intermedico). Extracted DNA was stored in a solution of 10 mmol/L Tris; 1 mmol/L EDTA, (pH 7.0–8.0;) and kept at -20°C. Prior to rTL analyses DNA from the two pinch biopsies was pooled and was quantified using by UV absorbance at 260 nm and diluted with molecular grade water (VWR International) to a concentration of 2 ng/μL.

rTL was measured by quantitative PCR (qPCR) using the method developed by Cawthon with several minor modifications [64]. Telomere repeat number and single copy gene number (albumin) were measured in separate qPCR reactions using a Bio-Rad CFX96 thermocycler (Bio-Rad Laboratories). Telomere primers (sequences described by Lan *et al*. [65]) and albumin primers (sequences described by Cawthon [66]) were manufactured by Integrated DNA Technologies. The qPCR master mix contained: 0.75 X SYBR Green I (Life Technologies); 10 mmol/L Tris HCl, pH 8.3 (Teknova); 50 mmol/L KCl (Sigma-Aldrich); 1 mol/L betaine (Sigma-Aldrich); 3 mmol/L MgCl_2_ (Sigma-Aldrich); 1 mmol/L DTT (Sigma-Aldrich); 0.2 mmol/L each dNTP (Life Technologies), 0.025 U/uL AmpliTaq Gold polymerase (Life Technologies) and either 500 nM telomere primers, or 300 nM albumin primers. Using 96-well plates, 15 μL of master mix was added to 20 ng of DNA in wells, such that the final qPCR reaction volume was 25 μL. Thermal cycling followed the protocol described by Lan *et al*. [65]. Melting curve analysis for each run confirmed amplification of the appropriate PCR products.

LinRegPCR software (2014 version) was used to determine C_q_ values of all samples and amplification efficiencies of both the telomere and the albumin (single copy gene) product [67]. qPCR efficiency, calculated for each plate, was used in the quantification of rTL to compensate for inter-plate variations in qPCR amplification. All samples were analysed in triplicate on 96-well plates. The acceptable standard deviation for the quantitation cycles (C_q_) for each sample was ≤ 0.3 [68].

Telomere length was expressed as a relative T/S ratio. The quantity of telomere (T) product was compared to the amount of albumin (S) product in each sample and were determined relative to a reference DNA sample using the Pfaffl [69] method. The reference DNA sample used to estimate rTL consisted of pooled leukocyte DNA from a 49 year old male and 29 year old female. Reference DNA was run on every plate. rTL values were corrected for inter-assay variability using a normalizing factor. A control sample (a commercially available sample of pooled leukocyte DNA from five males, Promega) was included on every plate and its average rTL across all runs was calculated. For each plate a normalization factor was calculated by dividing the rTL of the control sample on that plate by the average rTL for that same control sample. All rTL values were adjusted by dividing relative T/S ratios by plate specific normalization factors. Intra-assay coefficient of variation was 6.5% for the telomere assay and 4.3% for the albumin assay. Inter-assay coefficient of variation for rTL was 9.8%.

### Statistical analysis

Means and frequencies of selected characteristics for cases and controls were calculated and compared using *t-*tests or χ^2^ tests. To examine the association between rTL and colorectal adenoma prevalence, rTL was categorized into quartiles based on the rTL distribution among the controls. Logistic regression was used to examine the relationship between rTL (represented both as a continuous and categorical variable) and the prevalence of colorectal adenomas. To assess the possibility of a U-shaped relationship, a quadratic term of rTL was included in the model. Model fit was assessed using the likelihood ratio test. For all analyses in which rTL was represented as a continuous variable, rTL was standardized for ease of interpretation and to minimize collinearity in regression models with higher order terms. Factors related to adenoma risk (age, sex, BMI, smoking, alcohol consumption and NSAID use) were assessed in order to describe the study population. The main analyses did not adjust for any of these factors as they were considered upstream of telomere length in the hypothesized causal model. However, results of analyses adjusted for age are also presented. SAS software version 9.4 (SAS Institute Inc.) was used to conduct all statistical analyses.

## Results

The characteristics of the study population are shown in Table 1. In this study, prevalence of colorectal adenoma was significantly related to being male (*p* = 0.02), smoking (*p* = 0.004), and weakly related to older age (*p* = 0.13) and the use of non-steroidal anti-inflammatory drugs (*p = 0*.*08*). No relationship was observed between colorectal adenoma prevalence and obesity, or alcohol consumption. There was a weak inverse correlation between age and colon tissue rTL (*r* = -0.24, *p* = 0.03).

**Table 1:** Characteristics of the study population.

Characteristic	Outcome						*P value*^*a*^
	Normal			Adenoma			
	n	Mean (SD)	%	n	Mean (SD)	%	
Sex							0.02
Female	26		57.8	13		32.5	
Male	19		42.2	27		67.5	
Age, years							0.13
40–49	4		8.9	6		15.0	
50–59	34		75.6	22		55.0	
60–65	7		15.5	12		30.0	
Body mass index^b^	45	28.7 (5.5)		40	28.6 (5.4)		0.9
Normal	13		28.9	10		25.0	0.7
Overweight	16		35.6	18		45.0	
Obese	16		35.6	12		30.0	
Smoking							
Pack years^c^		0 (1.3)			3.8 (38.8)		0.004
Daily smoking							0.004
Ever	13		28.9	24		40.0	
Never	32		71.1	16		60.0	
Alcohol consumption							0.3
Low	38		84.4	30		75.0	
High	7		15.6	10		25.0	
Regular NSAID use							0.08
Yes	18		40.0	9		22.5	
No	27		60.0	31		77.5	
rTL	45	0 (1.0)		40	0.69 (1.34)		0.008

Abbreviations: SD, standard deviation; NSAID, non-steroidal anti-inflammatory drug

^a^ *P* for difference between participants with normal and adenoma outcomes, based on a 2-tailed *t*test, or χ^2^ test

^b^ Weight (kg) / height (m^2^)

^c^ Values presented are median (interquartile range) because the variable’s distribution was not normal

**Fig 1:**
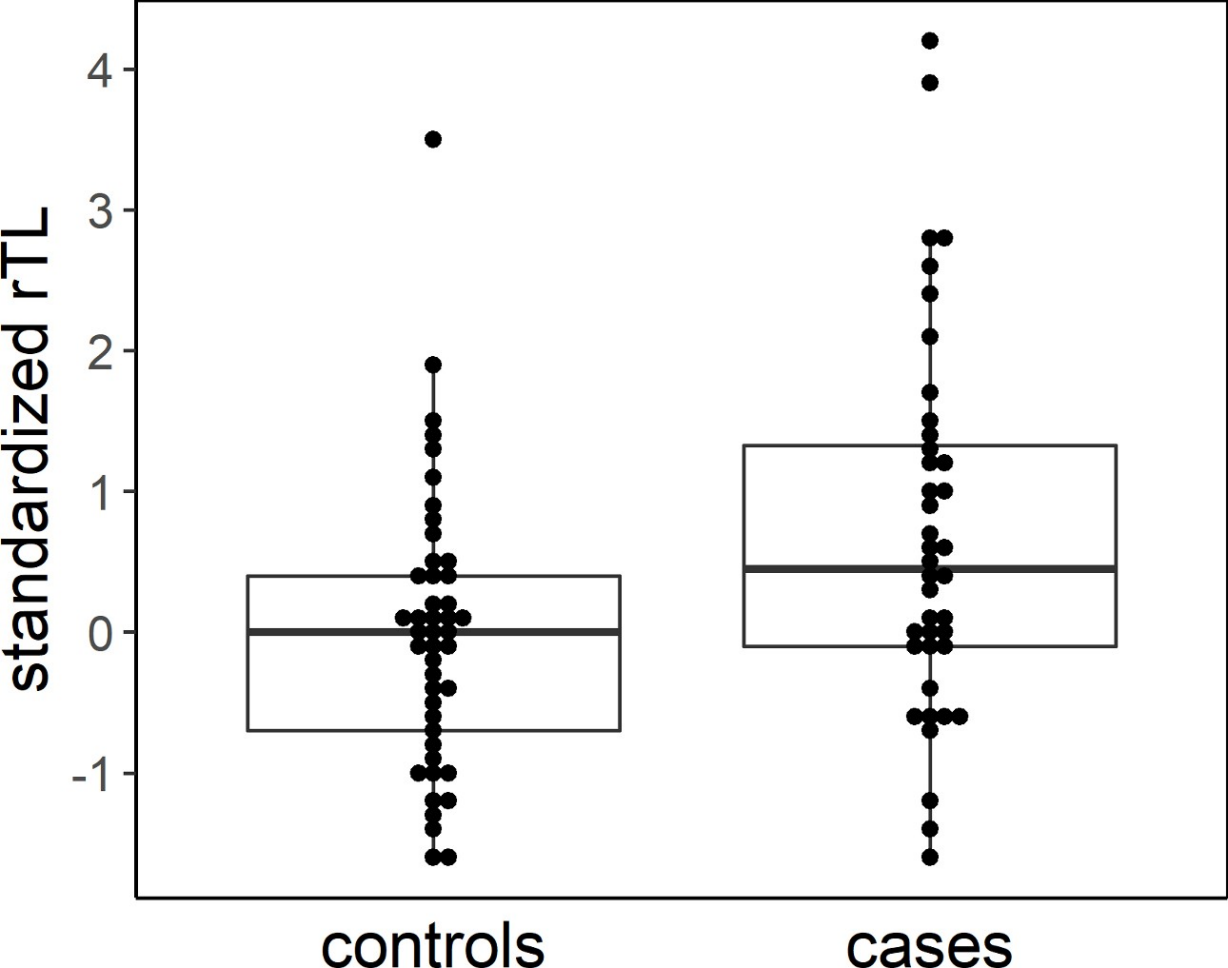
Beeswarm plot with overlying box plot showing the distribution of rTL among cases and controls.

A significant relationship between rTL in colon tissue and adenoma prevalence was observed. The distribution of rTL in controls was found to be significantly different from that of cases (Fig 1): the mean rTL in the colon tissue of adnoma cases was significantly higher than that of controls (*p* = 0.008). Analyses of the relationship between rTL quartile and adenoma prevalence (Table 2) indicated that individuals with the longest telomeres length had increased odds of adenoma when compared to the group with shortest telomere lengths (OR = 4.58, 95% CI:(1.19, 17.7)). Adjusting for age did not affect the magnitude of the observed relationship.

**Table 2:** ORs and 95% confidence intervals for the relationship between telomere length and colorectal adenoma prevalence.

Telomere length	No. Normal	No. Adenoma	OR (95%CI)	aOR^a^ (95%CI)
Quartile				
1 (short)	11	4	1.00 (reference)	1.00 (reference)
2	11	9	2.25 (0.53, 9.5)	2.28 (0.54, 9.7)
3	11	7	1.75 (0.40, 7.7)	1.74 (0.39, 7.7)
4 (long)	12	20	4.58 (1.19, 17.7)	4.86 (1.24, 19.1))
Overall^b^			1.69 (1.12, 2.55) *p =* 0.007	1.74 (1.15, 2.65) *p* = 0.005

^a^ Odds ratio adjusted for age

^b^ Logistic regression modelling colorectal adenoma prevalence on standardized rTL, odds ratio for a one standard deviation change

The shape of the relationship between telomere length and adenoma prevalence was found to be linear. Logistic regression models with rTL as a continuous variable indicated a positive dose-response relationship (Table 2). Inclusion of a quadratic term in the model to test for a U-shaped association between adenoma prevalence and telomere length was not significant (*p =* 0.9, likelihood ratio test). Odds of colorectal adenoma prevalence were lowest for those with the shortest rTL. Compared to individuals with average rTL (standardized rTL = 0), those with rTL one standard deviation above the mean (standardized rTL = 1) had a 69% increase in the odds of colorectal adenoma prevalence (*p =* 0.007). Results presented are not adjusted for covariates; adjustment for age did not meaningfully change results.

## Discussion

This nested case-control study assessed the relationship between colon tissue rTL and the prevalence of colorectal adenomas. Results from this study indicated a linear relationship between rTL in normal appearing colon tissue and the prevalence of colorectal adenomas. Compared to those with average rTL, those longer rTL had increased odds of having a colorectal adenoma. The odds ratios observed in this study provide evidence of direction, strength and statistcial significance of the relationship between rTL and colorectal adnoma risk, however these odds ratios do not provide an estimate for the prevalence risk ratio or incidence ratio.

Similar to what has been reported elsewhere, a weak negative correlation was observed between colon tissue rTL and age in this study. The relationship between age and rTL appears to be generally consistent across a number of tissues [4,5,70–73].

Epidemiologic evidence on the relationship between colon tissue telomere length and colorectal adenoma prevalence is limited. To the best of our knowledge, only one other study has measured telomere length in normal colon tissue of both patients with adenomas or cancers and healthy controls [40]. Fernandez-Rozadilla *et al*. [40] also reported an increased risk for colorectal cancer for the highest versus the lowest quartile of telomere length (OR = 1.9). Despite some differences in study design (age distribution of the study populations; composition of the case groups; and types of colon tissue in which telomere length was measured), the results of these two studies provide consistent evidence that telomere length has a significant role in colorectal cancer etiology.

The relationship between telomere length and colorectal risk has largely been studied by measuring telomere length in peripheral blood leukocytes and there is considerable heterogeneity in results across studies [30]. Prospective studies of this relationship have consisted of three case-control studies, nested within existing large cohorts, which have measured telomere length in stored blood samples collected at recruitment [37–39]. In contrast to the findings of the present study, results of prospective studies have not shown any evidence of a relationship between telomere length and risk of colorectal cancer. Some traditional case-control studies have shown short telomeres are associated with increased risk of colorectal cancer [39,41,42]; yet, other case-control studies have shown that both short and long telomeres are related to increased colorectal cancer risk [43,44]. Collectively, these results indicate that the relationship between telomere length and colorectal cancer risk may be complex and suggest that telomere length, at least as measured in peripheral blood leukocytes, is not strongly related to colorectal cancer risk years later [30]. Telomere dynamics in colonocytes differ from those in other tissues, specifically those of peripheral blood leukocytes [47]. The results of the present study suggests that telomere length in colon tissue prior to cancer development is associated with colorectal cancer risk. To improve understanding of telomere dynamics in the etiology of colorectal cancer it is important to have measures of telomere length in colon tissue.

This study was limited by a small sample size. The larger study from which cases and controls were selected included 334 participants who were eligible for this study, but due to resource contstraints we were only able to measure telomere length in the colon tissue of 85 individuals. As a result of the small sample size, confidence intervals for estimates were wide. Future studies with large sample sizes are needed to validate these results. Nevertheless, the results of this study are biologically plausible.

Traditional risk factors of colorectal cancer are thought to be upstream of telomere length in the causal pathway and therefore were not considered potential confounders in this study. A sensitivity analysis which evaluated age, sex, BMI, alcohol consumption and pack years of smoking as covariates in the statistical model, found only sex and pack years of smoking to be important covariates (using a liberal *p*-value of 0.15). Adjustment for covariates did not result in substantial changes to the estimated effect of rTL on adenoma prevalence and the conclusions remained the same. Previous studies have suggested that the relationship between telomere length and adenoma risk is modified by age and sex [30,42,44,47]. Given the small sample size in this study, it was not possible to test for interactions.

A number of sources of measurement error may have influenced the results of this study. Measurement of telomere length by qPCR is prone to random error. However, in this study rigourous quality controls were put in place to minimize qPCR measurement error as much as possible. In this study, rTL was measured in colonic tissue obtained from biopsies which likely contained a variety of cell types in which telomere dynamics may be different. The detection of colorectal adenomas during colonoscopy is not perfect, small adenomas (less than one centimetre in diameter) can easily be missed. The miss rate of adenomas is generally thought to be no more than 10%. Failure to detect adenomas among colonoscopy patients may have resulted in individuals being wrongly classified as having a normal colonoscopy and may have biased the results of this study. The detection rate of adenomas in this study is expected to be high as all colonoscopies were performed at a single clinical centre by experienced academic gastroenterologists whose detection rate is well within the acceptable range.

It is important to consider the time frame during which telomere length may affect the development of colorectal cancer. Currently, it is not known at which point(s) during the adenoma-carcinoma sequence telomere length may pose a risk. An advantage of using colorectal adenoma, rather than adenocarcinoma as an endpoint, is that the time period from exposure and the occurrence of the outcome is shortened; this shortened time frame may allow a relationship to be more easily detected. It was assumed that telomere length is relatively stable, such that measuring telomere length at time of adenoma diagnosis would represent an appropriate exposure window. It is unlikely that adenoma would affect telomere length in distant normal appearing tissue, so reverse causation is not a concern. However, the use of adenoma prevalence as an outcome has the potential to cause bias. Prevalence is a function of incidence and duration, so the prevalence odds ratios of the relationship between rTL and colorectal adenomas may be subject to incidence prevalence bias. It is possible that telomere length affects the length of time for progression through the adenoma-carcinoma sequence. If this were the case, the results from this study would be biased.

Given what is known about the important role of telomere length in chromosomal stability and regulating cellular proliferation, it is plausible that long telomeres in colon tissue increase the risk of colorectal cancer. Our results, which indicate that increased telomere length is related, in a dose-response manner, to an increased prevalence of colorectal adenomas, strengthen the conclusions of the only other paper which has addressed the same research question with a similar study design [40]. Nevertheless, the role of telomere length in colorectal cancer etiology needs to be further characterized.

## Supporting information

S1 Table Participant characteristics and rTL data. (XLSX)
